# Enhancing Ultrasound Image Quality Across Disease Domains: Application of Cycle-Consistent Generative Adversarial Network and Perceptual Loss

**DOI:** 10.2196/58911

**Published:** 2024-12-17

**Authors:** Shreeram Athreya, Ashwath Radhachandran, Vedrana Ivezić, Vivek R Sant, Corey W Arnold, William Speier

**Affiliations:** 1 Department of Electrical and Computer Engineering University of California Los Angeles Los Angeles, CA United States; 2 Department of Bioengineering University of California Los Angeles Los Angeles, CA United States; 3 Medical Informatics University of California Los Angeles Los Angeles, CA United States; 4 Department of Surgery The University of Texas Southwestern Medical Center Dallas, TX United States; 5 Department of Radiological Sciences University of California Los Angeles Los Angeles, CA United States; 6 Department of Pathology and Laboratory Medicine University of California Los Angeles Los Angeles, CA United States

**Keywords:** generative networks, cycle generative adversarial network, image enhancement, perceptual loss, ultrasound scans, ultrasound images, imaging, machine learning, portable handheld devices

## Abstract

**Background:**

Numerous studies have explored image processing techniques aimed at enhancing ultrasound images to narrow the performance gap between low-quality portable devices and high-end ultrasound equipment. These investigations often use registered image pairs created by modifying the same image through methods like down sampling or adding noise, rather than using separate images from different machines. Additionally, they rely on organ-specific features, limiting the models’ generalizability across various imaging conditions and devices. The challenge remains to develop a universal framework capable of improving image quality across different devices and conditions, independent of registration or specific organ characteristics.

**Objective:**

This study aims to develop a robust framework that enhances the quality of ultrasound images, particularly those captured with compact, portable devices, which are often constrained by low quality due to hardware limitations. The framework is designed to effectively process nonregistered ultrasound image pairs, a common challenge in medical imaging, across various clinical settings and device types. By addressing these challenges, the research seeks to provide a more generalized and adaptable solution that can be widely applied across diverse medical scenarios, improving the accessibility and quality of diagnostic imaging.

**Methods:**

A retrospective analysis was conducted by using a cycle-consistent generative adversarial network (CycleGAN) framework enhanced with perceptual loss to improve the quality of ultrasound images, focusing on nonregistered image pairs from various organ systems. The perceptual loss was integrated to preserve anatomical integrity by comparing deep features extracted from pretrained neural networks. The model’s performance was evaluated against corresponding high-resolution images, ensuring that the enhanced outputs closely mimic those from high-end ultrasound devices. The model was trained and validated using a publicly available, diverse dataset to ensure robustness and generalizability across different imaging scenarios.

**Results:**

The advanced CycleGAN framework, enhanced with perceptual loss, significantly outperformed the previous state-of-the-art, stable CycleGAN, in multiple evaluation metrics. Specifically, our method achieved a structural similarity index of 0.2889 versus 0.2502 (*P*<.001), a peak signal-to-noise ratio of 15.8935 versus 14.9430 (*P*<.001), and a learned perceptual image patch similarity score of 0.4490 versus 0.5005 (*P*<.001). These results demonstrate the model’s superior ability to enhance image quality while preserving critical anatomical details, thereby improving diagnostic usefulness.

**Conclusions:**

This study presents a significant advancement in ultrasound imaging by leveraging a CycleGAN model enhanced with perceptual loss to bridge the quality gap between images from different devices. By processing nonregistered image pairs, the model not only enhances visual quality but also ensures the preservation of essential anatomical structures, crucial for accurate diagnosis. This approach holds the potential to democratize high-quality ultrasound imaging, making it accessible through low-cost portable devices, thereby improving health care outcomes, particularly in resource-limited settings. Future research will focus on further validation and optimization for clinical use.

## Introduction

Ultrasound imaging is crucial in medical diagnostics due to its noninvasive nature and high accuracy. It provides point-of-care assessments that have been increasingly adopted by health care professionals [1,2]. Historically, technology has been limited to large, expensive devices typically found in specialized medical settings. However, there has been a transformative shift toward the development and adoption of compact, handheld ultrasound devices [3,4]. These smaller devices promise to democratize access to medical imaging by making it more affordable and widely available. Yet, the miniaturization and cost-effectiveness often come at the expense of image quality, a trade-off primarily attributable to hardware constraints [5-7].

Machine learning algorithms have been explored to enhance low-quality images without the need for hardware improvements [8]. For instance, generative adversarial networks (GANs) [9] have been used to create high-quality reconstructions of ultrasound images and videos, providing a cost-efficient avenue for the enhancement of portable ultrasound devices [10-12]. The cycle-consistent generative adversarial network (CycleGAN) framework, which is particularly useful for image-to-image translations without requiring paired data, has become increasingly popular [13]. The technology has been applied across a spectrum of tasks including, style transfer [14], where the appearance of one image is transformed to match another style, and object transfiguration [13,15,16], which involves changing 1 object in an image into another while retaining the overall structure. In medical imaging, CycleGANs have been used in tasks such as pixel-wise translation in echocardiography [17]. CycleGANs have also been applied in cross-modality medical image translation such as converting computed tomography to magnetic resonance imaging [18]. The architecture has even found use in histopathology to standardize microscopy staining for more accurate diagnoses [19].

We hypothesize that the integration of computational algorithms, particularly CycleGAN, can mitigate the disparities in images acquired from different medical imaging devices. Traditional training approaches for these models artificially introduce corruption into medical images to create pixel-wise pairs [20-22]. However, these methods typically fail to encapsulate the different characteristics of images acquired using different devices. Acquiring paired images using different devices leads to technical issues as images are captured at different time instances with varying orientations, leading to structural changes that cannot be completely resolved using image registration.

In this work, we benchmark several key models that are highly relevant to our task of ultrasound image enhancement. Pix2Pix [4] uses conditional adversarial networks for paired image-to-image translation, making it effective for directly comparing low- and high-quality images. CycleGAN [5] enables unpaired image-to-image translation, which is crucial when paired datasets are not available. Registration GAN (RegGAN) [6] focuses on medical image translation by aligning structural content using a registration network, and multilevel structure-preserved GAN (MSPGAN) [7] introduces a multilevel structure-preserved GAN for domain adaptation in intravascular ultrasound analysis. However, the current state-of-the-art is the stability-enhanced CycleGAN [1], which specifically addresses domain transformation challenges in unpaired ultrasound images, making it particularly relevant and effective for our specific application.

Evaluation metrics play a critical role in assessing the effectiveness of image enhancement models. Commonly used metrics include structural similarity index (SSI), peak signal-to-noise ratio (PSNR), and locally normalized cross-correlation (LNCC) [5,10-12,23]. While these metrics are widely accepted, they primarily capture low-frequency information and may not adequately reflect true image quality, particularly in preserving high-frequency details, which are crucial for medical diagnostics. Models that perform well on these traditional metrics may produce visually appealing images but fail to retain essential high-frequency content, leading to a loss of critical diagnostic information and perceptual quality [24]. To address this limitation, we incorporate the learned perceptual image patch similarity (LPIPS) [24] metric in our evaluations. LPIPS is designed to capture perceptual differences that align more closely with human visual perception, ensuring that our method not only performs well quantitatively but also produces qualitatively superior images, preserving both low- and high-frequency details essential for accurate medical analysis.

To overcome these challenges, our approach leverages perceptual loss, which can eliminate the need for registration and more accurately relate images from disparate domains. Traditional loss functions used in CycleGAN can result in hallucinated features in the enhanced images [25]. By incorporating perceptual loss [24], more interpretable images are generated that are more robust to registration artifacts [26]. This method can enhance the reliability and consistency of images from handheld ultrasound devices to bridge the gap with expensive high-end systems for greater equity in access to health care.

## Methods

### Study Design

In this study, we aim to address the challenge of enhancing ultrasound image quality, particularly for images captured by compact, portable devices that often suffer from lower quality due to hardware limitations. To achieve this, we used a CycleGAN framework enhanced with perceptual loss. This approach focuses on processing nonregistered image pairs from various organ systems, ensuring that the enhanced images retain anatomical integrity and closely mimic high-resolution outputs. Our method is designed to be robust, versatile, and applicable across diverse clinical settings.

### Model Overview

**Figure 1:**
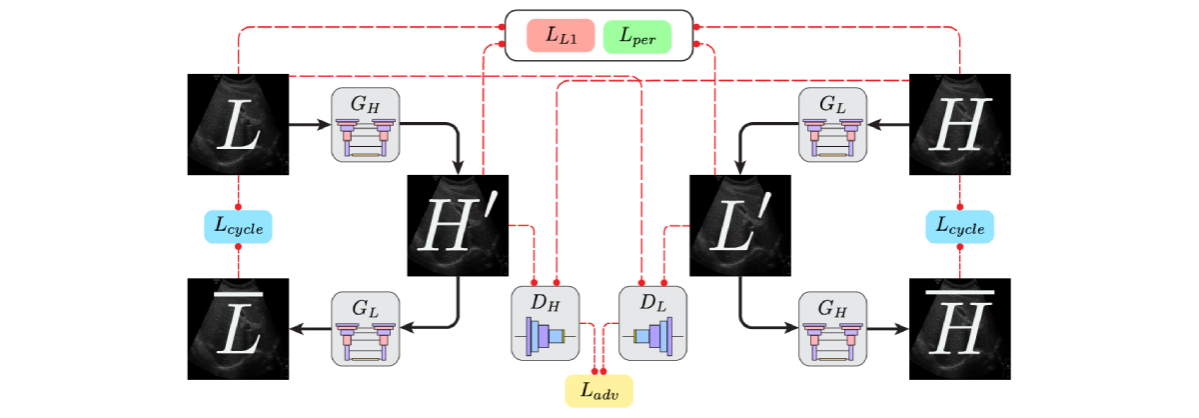
An overview of the cycle generative adversarial network model training and loss computation framework. The solid black arrows indicate the flow of data. The dashed red arrows indicate the flow of information for loss computation.

Our framework for generating high-quality images is a modification of the CycleGAN architecture, designed to map between 2 distinct imaging domains. In ultrasound image enhancement, these domains correspond to low-quality (domain *L*) and high-quality (domain *H*) images. The model uses 2 generators, *G_L_* and *G_H_*, and 2 discriminators, *D_L_* and *D_H_* (Figure 1). Note that the generators *G_L_* and *G_H_* share the same model architecture. Similarly, the discriminators *D_L_* and *D_H_* share the same model architecture. The generator *G_L_* is responsible for converting an image from domain H, which represents high-quality images, to domain L, characterized by low-quality images. Conversely, the generator *G_H_* performs the opposite transformation, taking an image from domain L and converting it to align with domain H. This bidirectional transformation process is essential for the task of image enhancement, as it allows for the improvement of low-quality images by translating them into their high-quality counterparts. The discriminators aim to distinguish real images in their respective domains from those transformed by the generators. A unique feature of this approach is the cycle consistency loss [13], which plays a crucial role in image quality enhancement. This loss ensures that when an image is translated to the other domain and then reverted to its original domain, it closely resembles the original image. Specifically, for enhancing low-quality images to high-quality images, the cycle consistency loss maintains the integrity of the image content throughout the transformation process. This prevents the introduction of artifacts and ensures that the enhanced image retains the essential features of the original low-quality image, resulting in a high-quality output that remains true to the source. After training, the *G_H_* generator is used to enhance images, maintaining essential structural attributes while improving clarity and resolution.

### Model Description

GANs have seen transformative advancements, with CycleGAN [13] representing a significant milestone in facilitating unsupervised image-to-image translations. The GAN architecture comprises 2 primary modules: the generator and the discriminator.

**Figure 2:**
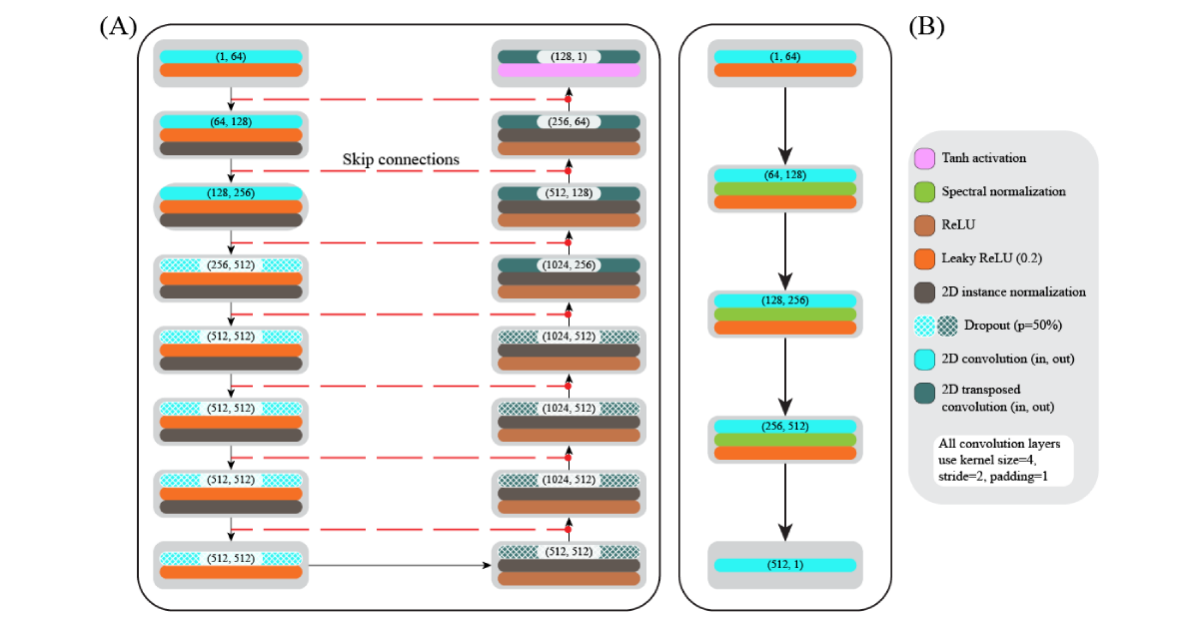
The model architectures. The (A) generator and (B) discriminator model architectures. The figure legend lists the different layers in the models. ReLU: rectified linear unit.

The generator (Figure 2A) architecture is inspired by the generator used by Isola et al [27]. The generator network is structured as a UNet [28], divided into encoding and decoding phases, incorporating detailed mechanisms for both down-sampling and up-sampling the input data. The encoder initiates with a 64-channel 2D convolutional layer designed to capture broad contextual details. This phase uses multiple down-sampling layers, each comprising a convolutional layer with instance normalization and leaky rectified linear unit (ReLU) activation functions. The instance normalization layers stabilize the training process by normalizing the feature maps, while leaky ReLU activations introduce nonlinearity and mitigate the vanishing gradient problem. To enhance model robustness, dropout layers are included in deeper layers of the encoder. The down-sampling process reduces the spatial dimensions while increasing the depth, thereby emphasizing hierarchical feature extraction. In the decoding phase, the model uses transposed convolutional layers for up-sampling, which restores the spatial dimensions. Each up-sampling step involves skipping connections from the corresponding down-sampling layers, preserving detailed features from earlier stages. These layers also incorporate instance normalization and ReLU activations, where ReLU functions introduce nonlinearity, promoting sparse activations and efficient learning. The final layer uses a tanh activation function, scaling the output values to [–1, 1], suitable for image generation tasks. This design ensures effective image enhancement by maintaining high-quality feature extraction and reconstruction.

The discriminator (Figure 2B) distinguishes between real and generated images. We use spectral normalization [29] to ensure stability during training. Its architecture begins with a convolutional layer that compresses spatial information and expands depth followed by a leaky ReLU activation layer. Subsequent layers maintain the use of spectral normalization to ensure 1 – Lipschitz continuity. This constraint on the spectral norm of each layer’s weights helps to balance the generator and discriminator during training. The PatchGAN [27,30] style discriminator output is a 30×30 grid with a depth of 1, which provides a spatial map indicating the likelihood of each region in the input image being real or generated. This final classification output allows for a more detailed evaluation of the image, helping to distinguish between authentic and synthetic content across different spatial locations.

### Loss Function

#### Perceptual Loss

Conventional methodologies like mean squared error (MSE) and SSI rely on pixel-wise alignment which makes them unsuitable for nonregistered image pairs acquired using different devices. The LPIPS metric addresses these constraints by evaluating the perceptual similarity between images [24]. LPIPS leverages deep features extracted from pretrained convolutional networks, such as the visual geometry group network [31]. The LPIPS metric comparing images *X* and *Y* is given by the following equation.



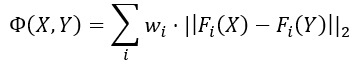



where *F*_i_ and *w*_i_ denotes the feature maps and optimized weights from the *i*^th^ layer of the pretrained network. Deep feature maps are systematically extracted from every layer within the network, ensuring a comprehensive reflection of the multi-scale characteristics of human perceptual judgment. These features are then unified through linear combination, optimizing the weights to align with perceptual judgments assessed by human evaluators. The LPIPS metric consistently outranks traditional metrics, showcasing superior performance across an array of perceptual judgment tasks [26]. This loss is calculated between real images *L* and *H*, and those generated through the CycleGAN framework’s generators as follows.



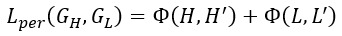



where *H^'^* represents *G_H_(L)* and *L^'^* represents *G_L_(H)*.

#### Generator Loss

The generator’s loss function is a linear combination of several distinct loss terms, each playing a pivotal role in optimizing image translation between the 2 domains. First, adversarial loss *L_adv_* (*D_H_,D_L_*) induces the discriminators to perceive generated images as genuine, whether they are translated from low to high quality or vice versa.

*L_adv_* (*D_H_,D_L_*) = *MSE*(*1,D_H_*) + *MSE*(*1,D_L_*)

Specifically, MSE calculates the discrepancy between the discriminator’s predictions and an array of ones. The array of ones represents the target output for real images, indicating that the discriminator should classify these images as genuine. By comparing the discriminator’s predictions to this ideal output, the MSE helps measure how far the generated images are from being perceived as real. These terms push the generator to produce images that can convince the discriminator they belong to the high-quality domain, thereby improving the realism and quality of the generated images. Using an array of ones ensures that the generator is continuously driven to reduce the difference between its output and real high-quality images, enhancing its performance over time. The cycle loss *L_cycle_* (*G_H_,G_L_*) prevents the loss of critical image features by ensuring that an image translated to the other domain and back yields the original image.



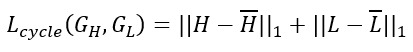



where 
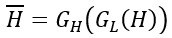
. and 
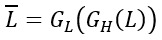
. The *L*1 loss *L_L_*_1_(*G_H_*,*G_L_*) ensures that the generated images are closer to true high-quality images in an *L*1 distance sense.



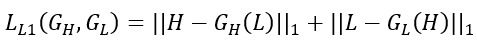



Finally, the aggregate generator loss, *L*(*G_H_,G_L_,D_H_,D_L_*), is computed by combining all individual loss terms weighed by their respective lambda constants.



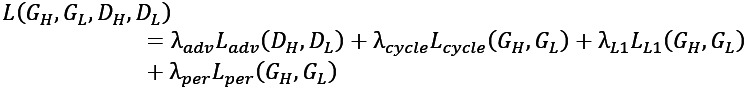



By using this multifaceted loss function, the model ensures that the generators achieve high-quality image translations while preserving the intrinsic characteristics of the source domain.

#### Discriminator Loss

The discriminator loss function is designed to evaluate the authenticity of images, incorporating the principle of label smoothing to further enhance the model’s generalizability. The discriminator is tasked with distinguishing between real images from the dataset and synthetic images generated by the corresponding generator.



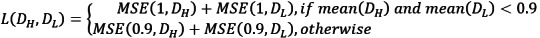



For each domain, the discriminator computes scores for both real and synthetic images. Conventionally, discriminators are trained using hard labels, where real images are labeled as “1” and synthetic images as “0.” However, hard labels can cause vulnerability to adversarial perturbations—small, intentional changes to the input that can deceive the model into making incorrect predictions—and lead to overconfidence, where the discriminator becomes excessively certain in its predictions. Label smoothing improves the generalization and robustness of neural networks by preventing overconfidence in predictions. Szegedy et al [32] demonstrated its effectiveness in reducing overfitting and enhancing performance in image classification. Similarly, Salimans et al [33] applied 1-sided label smoothing in the training of GANs, which helped stabilize training and improve the quality of generated images. These studies support the use of label smoothing as a strategy to mitigate the negative effects of hard labels. In our framework, if the mean scores of both real and synthetic images for the high-quality domain are less than 0.9, the label 1.0 is used. Otherwise, a smoothing factor of 0.9 is applied, meaning the real images are given a target value slightly less than 1, to prevent overconfidence and promote model robustness. The total discriminator loss, *L*(*D_H_*,*D_L_*), is then computed by aggregating the individual MSE losses 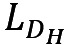. and 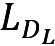. for high and low-quality domains, respectively.

### Implementation Details

All models were trained for 300 epochs with a batch size of 4 images. We used the Adam optimizer for model optimization, with a learning rate (3×10^–4^) set for both the generators and the discriminators. A beta value of 0.9 for the first and 0.999 for the second moments were used in each optimizer. A learning rate scheduler reduced learning rates by half (γ=.5) every 100 epochs, to allow adaptability during training. Weights were assigned to each loss term: λ_a_*_dv_*=1 for adversarial loss, λ*_cycle_*=10 for cycle-consistency loss, λ*_L_*_1_=2 for *L*1 loss, and λ*_per_*=10 for perceptual loss. The overall dataset was split with 70% for model training, 10% for model validation, and 20% for the hold-out test set. Gradient scaling was used to optimize the model’s precision and speed. Code implementation will be made publicly available.

### Evaluation

The synthetic high-quality images generated by the model are evaluated using 4 key metrics: SSI, LNCC, PSNR, and LPIPS. Each of these metrics provides a unique perspective on the fidelity and quality of the generated images compared to the ground truth.

- SSI evaluates the structural fidelity between the generated image *H^'^* and the actual high-quality image *H*. It considers 3 aspects: luminance, contrast, and structure. The SSI is computed as follows.

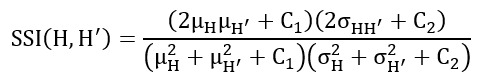



where µ*_H_* and µ*_H^'^_* are the mean intensities, 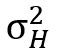 and 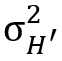 are the variances, and 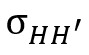 is the covariance between *H^'^* and *H*. The constants *C*_1_ and *C*_2_ are used to stabilize the division.

- LNCC measures the local similarity in intensity patterns between the generated and actual high-quality images. This metric is particularly sensitive to local differences in intensity, making it useful for detecting fine-grained discrepancies. LNCC is calculated by dividing the cross-correlation of local image patches by the product of their local SDs.

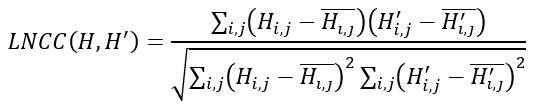



where 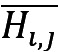. and 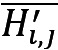. are the local means of the patches.

- PSNR quantifies the error signal strength between the generated and actual images, derived from the MSE. It is defined as follows.

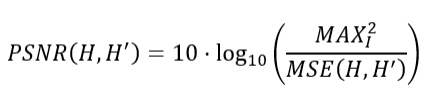



where *MAX_I_* is the maximum possible pixel value of the image, and *MSE (H, H^'^)* is the mean squared error between *H^'^* and *H*. A higher PSNR indicates that the generated image is closer to the high-quality reference.

- LPIPS [24] is a perceptual metric that compares deep features extracted from a neural network rather than directly comparing pixel values. This approach better aligns with human perception of image similarity. LPIPS is computed by passing both the generated and actual images through a pretrained deep network and measuring the distance between their respective feature representations. Lower LPIPS values indicate higher perceptual similarity.

Together, these metrics provide a comprehensive evaluation of the generated images, which is particularly relevant for our image enhancement task, where unregistered low-high quality image pairs are compared. SSI and LNCC assess how well the structural and intensity patterns are preserved during enhancement, even when images are not perfectly aligned. PSNR quantifies the reduction in error relative to the original image, indicating overall fidelity. LPIPS, on the other hand, evaluates perceptual quality, ensuring that the enhancement appears natural and realistic to human observers, even in challenging scenarios with misaligned inputs.

### Ethical Considerations

This study did not involve the collection of new data from human participants. The dataset used is publicly available and provided as part of the 26th International Conference on Medical Image Computing and Computer Assisted Intervention (MICCAI 2023) USEnhance Challenge [34,35]. Therefore, no ethics review or approval was required for this study. As the study used publicly available data provided by the organizers of the challenge, informed consent specific to this research was not required. It is assumed that the original informed consent for data collection includes provisions for secondary analysis without requiring additional consent. All images used in this study are fully deidentified with no personal health information included. The dataset provided by the challenge organizers ensured anonymity, thus protecting the privacy and confidentiality of any potential human participants. There was no direct interaction with human participants in this study; hence, no compensation was provided. No images in this study or its supplementary materials allow for the identification of individual participants. All data are deidentified and anonymous, ensuring that no individual can be recognized from the images used.

## Results

### Overview

This study uses a dataset consisting of 2100 ultrasound images, including 1050 pairs of low- and high-quality images (Table 1 [34]). These images were collected from 131 patients with suspected thyroid tumors, carotid plaque, or breast cancer, along with healthy participants. During scans, volunteers were instructed to hold their breath for approximately 10 seconds to minimize deformation, and landmark points were noted for nonrigid registration to ensure the creation of accurate data pairs. This well-curated dataset provides a robust foundation for this study. This dataset was provided by the organizers of the MICCAI 2023 USEnhance Challenge [34]. Our baseline compares low-quality images directly to high-quality images without any enhancement or learning-based processing, serving as the starting point for evaluating the effectiveness of various models, including our approach. The models benchmarked in this study include Pix2Pix [27], MSPGAN [11], CycleGAN [13], RegGAN [23], and stable CycleGAN [12]. Among these, MSPGAN, RegGAN, and stable CycleGAN are the most recent advancements and are considered state-of-the-art for this task. To rigorously assess the improvements offered by our method, we computed the statistical significance of our results using the 1-sided Wilcoxon signed rank test.

**Table 1:** Dataset summary across different ultrasound devices and organs.

Organ	Low-end device	High-end device	Patients (n=131), n (%)	Ultrasound image pairs, n (%)		
				Training (n=840)	Testing (n=210)	Total (n=1050)
Thyroid	mSonics MU1	Toshiba Aplio 500	33 (25.2)	233 (27.7)	58 (27.6)	291 (27.7)
Carotid	SSUN	Toshiba Aplio 500	54 (41.2)	229 (27.3)	57 (27.1)	286 (27.2)
Abdomen	SSUN	General Electric LOGIQ E9	21 (16)	217 (25.8)	54 (25.7)	271 (25.8)
Breast	mSonics MU1	Aixplorer ultrasound system (SuperSonic Imaging SA)	23 (17.6)	161 (19.2)	41 (19.5)	202 (19.2)

### Quantitative Results

In the evaluation of the SSI, our proposed method achieved a score of 0.2889 (Table 2), surpassing the reference low baseline (0.2363; *P*<.001), as well as CycleGAN (0.2622; *P*<.001) and stable CycleGAN (0.2502; *P*<.001). This places our method on par with the top-performing models like Pix2Pix (0.2862; *P* *P*>.99), MSPGAN (0.2796; *P*<.001), and RegGAN (0.2809; *P*<.001). Among the methods evaluated, stable CycleGAN exhibited the lowest SSI score, indicating the least effective structural preservation. Pix2Pix, on the other hand, performed slightly better than MSPGAN and RegGAN, highlighting its strength in maintaining structural details. For LNCC, our method recorded a score of 0.8454, which is significantly higher than the reference low baseline (0.7836; *P*<.001) and comparable to the scores achieved by MSPGAN (0.8535; *P*>.99) and Pix2Pix (0.8491; *P*>.99). While MSPGAN led in LNCC, the differences between the top performing methods are minimal, underscoring the similar performance levels across these models. Notably, CycleGAN and stable CycleGAN scored 0.8271 (*P*<.001) and 0.8145 (*P*<.001), respectively, showing lower but still competitive performance.

In terms of PSNR, the proposed method achieved a score of 15.8935, which is a marked improvement over the reference low baseline (14.2978; *P*<.001). Although Pix2Pix (16.3914; *P*>.99) and MSPGAN (16.2602; *P*>.99) reported higher PSNR values, indicating lower overall error between the generated and high-quality images, the differences between these models and our approach are modest. RegGAN also performed well with a score of (16.2721; *P*>.99), while CycleGAN (14.9126; *P*<.001) and stable CycleGAN (14.9430; *P*<.001) had lower PSNR values, indicating higher error rates. Finally, for the LPIPS metric, our method demonstrated the best performance with a score of 0.4490, significantly lower than Pix2Pix (0.4664; *P*<.001), MSPGAN (0.4709; *P*<.001), and RegGAN (0.4855; *P*<.001). This indicates that our method produced images that were perceptually closer to high-quality outputs. CycleGAN and stable CycleGAN reported LPIPS scores of 0.4828 (*P*<.001) and 0.5005 (*P*<.001), respectively, with stable CycleGAN showing the least favorable performance among all models in terms of perceptual quality. Across these metrics, while certain models like Pix2Pix and MSPGAN excel in specific metrics such as LNCC and PSNR, our approach consistently delivers competitive performance, particularly in SSI and LPIPS, making it a robust framework for ultrasound image enhancement.

**Table 2:** Performance evaluation of models on the test set.

Model configurations	LNCC^a^↑^b^	SSI^c^↑	PSNR^d^↑	LPIPS^e^↓^f^
Reference low	0.7836^g^	0.2363^g^	14.2978^g^	0.5080^g^
Pix2Pix [27]	0.8491	0.2862	16.3914	0.4664^g^
MSPGAN^h^ [11]	0.8535	0.2796^g^	16.2602	0.4709^g^
CycleGAN^i^ [13]	0.8271^g^	0.2622^g^	14.9126^g^	0.4828^g^
RegGAN^j^ [23]	0.8475	0.2809^g^	16.2721	0.4855^g^
Stable CycleGAN [12]	0.8145^g^	0.2502^g^	14.9430^g^	0.5005^g^
Proposed method	0.8454	0.2889	15.8935	0.4490

^a^LNCC: locally normalized cross-correlation.

^b^↑: higher scores are better.

^c^SSI: structural similarity index.

^d^PSNR: peak signal-to-noise ratio.

^e^LPIPS: learned perceptual image patch similarity

^f^↓: lower scores are better.

^g^Statistically significant improvement.

^h^MSPGAN: multilevel structure-preserved generative adversarial network.

^i^CycleGAN: cycle-consistent generative adversarial network.

^j^RegGAN: registration generative adversarial network.

### Qualitative Results

**Figure 3:**
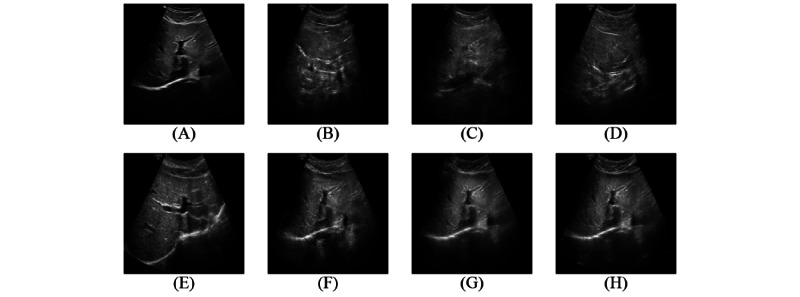
A comparative visualization of ultrasound scans from the test set, showcasing the performance of different enhancement frameworks on the same high-low quality image pair. Each subfigure corresponds to a different model’s output, allowing for a direct comparison of the anatomical preservation and image quality achieved by each approach. (A) reference low, (B) Pix2Pix [27], (C) MSPGAN [11], (D) RegGAN [23], (E) reference high, (F) CycleGAN [13], (G) stable CycleGAN [12], and (H) proposed method. CycleGAN: cycle-consistent generative adversarial network; MSPGAN: multilevel structure-preserved generative adversarial network; RegGAN: registration generative adversarial network.

**Figure 4:**
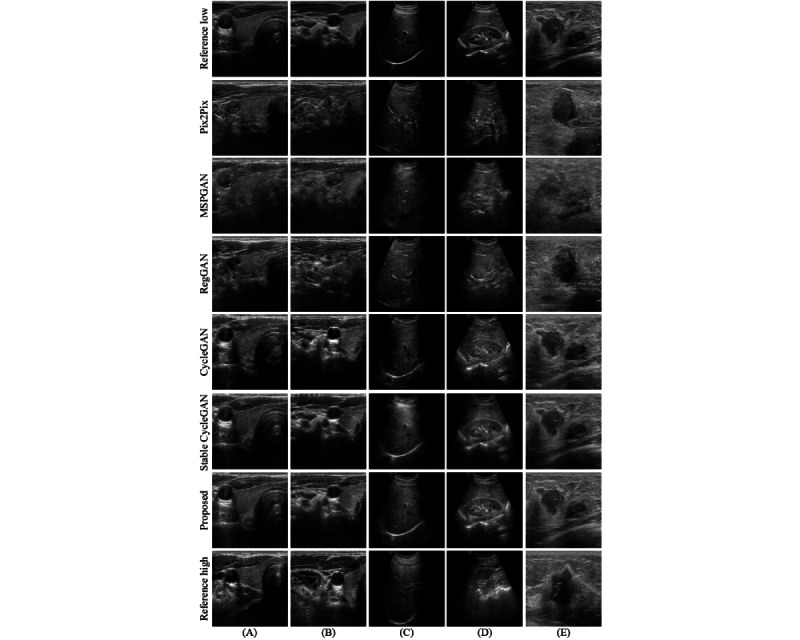
A comparative visualization of ultrasound scans from the test set, showcasing the performance of different enhancement frameworks on the same high-low quality image pairs. (A) Thyroid, (B) carotid, (C) liver, (D) kidney, and (E) breast. CycleGAN: cycle-consistent generative adversarial network; MSPGAN: multilevel structure-preserved generative adversarial network; RegGAN: registration generative adversarial network.

Qualitative analysis further illustrates the differences in the generated images across the models. As shown in Figures 3 and 4, methods such as Pix2Pix, MSPGAN, and RegGAN, despite their higher scores in SSI, LNCC, and PSNR, often produce images that lack anatomical detail and introduce distortions that may affect clinical interpretation. In contrast, methods like CycleGAN, Stable CycleGAN, and our proposed approach maintain the integrity of anatomical structures, ensuring that the generated images closely resemble the original high-quality images. Our approach is particularly effective in preventing the loss of critical diagnostic information, which is essential for accurate medical assessments. While quantitative metrics provide a useful evaluation framework, the qualitative results underscore the importance of preserving anatomical integrity, an area where our method excels.

## Discussion

In this study, we developed and evaluated an advanced CycleGAN framework enhanced with perceptual loss to address the challenge of varying image quality in ultrasound imaging across different devices. Our primary objective was to improve the quality of low-resolution ultrasound images captured by portable devices while preserving anatomical integrity, which is critical for accurate clinical diagnostics. The results from our evaluation demonstrated that the integration of perceptual loss enhanced the quality of the generated images, achieving strong performance in key metrics such as SSI and LPIPS, though slightly lower in LNCC and PSNR compared to other models. These outcomes suggest that our approach represents a significant step toward bridging the gap between low- and high-quality ultrasound images, making it particularly beneficial for portable, handheld devices that often struggle with image quality due to hardware limitations.

The use of perceptual loss in our model allowed for a more direct and meaningful comparison between low- and high-quality images, which contrasts with previous studies that treated these domains as independent [12]. By leveraging paired images from different devices, our model was able to learn the nuances of quality differences in a manner that closely mirrors real-world clinical scenarios. This pairing led to significant improvements in metrics such as SSI and LPIPS, indicating that our model preserves structural fidelity and local intensity patterns more effectively than current state-of-the-art approaches. However, it is important to note that while some models, such as Pix2Pix, MSPGAN, and RegGAN, achieve high scores in SSI, LNCC, and PSNR, they often do so at the expense of anatomical integrity. These models tend to remove or alter critical anatomical structures, leading to a loss of valuable diagnostic information. In contrast, our approach retains the anatomical content while producing comparable performance in these metrics and outperforming all other models in LPIPS, which measures perceptual quality. This balance between maintaining anatomical fidelity and achieving high image quality is a significant strength of our method, making it more suitable for clinical applications where accuracy is paramount.

Despite these promising results, there are some limitations to our approach that need to be addressed in future work. The reliance on perceptual loss, while beneficial for maintaining image fidelity, introduces additional computational complexity, leading to longer training times. This requirement could be a limitation in scenarios where computational resources are limited or rapid model deployment is necessary. Additionally, while our model has demonstrated strong performance across a well-curated dataset, the findings need to be validated through extensive real-world applications across diverse datasets and imaging conditions to ensure robustness and generalizability. Furthermore, the current model is designed to work across various organ systems and diseases, but future research could explore the development of more specialized models tailored to specific clinical contexts, potentially optimizing performance for targeted diagnostic tasks.

To conclude, this work introduced an advanced CycleGAN-based framework that effectively enhances ultrasound image quality across devices by using perceptual loss to train on paired images. Our findings demonstrate the feasibility of bridging the image quality gap between low- and high-quality ultrasound images, thereby improving the accessibility and equity of high-quality diagnostic imaging. As we move forward, it will be crucial to conduct clinical validation of this approach across a wide range of medical scenarios and explore its application to other imaging modalities. This result could pave the way for integrating our model into routine clinical practice, ultimately enhancing diagnostic accuracy and improving patient outcomes. By making high-quality imaging more accessible, particularly through portable ultrasound devices, our approach holds the potential to significantly impact health care delivery and patient care on a global scale.

## Abbreviations

CycleGAN: cycle-consistent generative adversarial network
GAN: generative adversarial network
LNCC: locally normalized cross-correlation
LPIPS: learned perceptual image patch similarity
MICCAI: Medical Image Computing and Computer Assisted Intervention
MSE: mean squared error
MSPGAN: multilevel structure-preserved generative adversarial network
PSNR: peak signal-to-noise ratio
RegGAN: registration generative adversarial network
ReLU: rectified linear unit
SSI: structural similarity index

## Data Availability

The datasets generated during and/or analyzed during this study are available in the MICCAI (Medical Image Computing and Computer Assisted Intervention) 2023 USEnhance Challenge repository [34].
